# A scintillation dosimeter with real‐time positional tracking information for in vivo dosimetry error detection in HDR brachytherapy

**DOI:** 10.1002/acm2.14150

**Published:** 2023-09-20

**Authors:** Daline Tho, Marie‐Claude Lavallée, Luc Beaulieu

**Affiliations:** ^1^ Department of Radiation Oncology The University of Texas MD Anderson Cancer Center Houston Texas USA; ^2^ Département de physique, de génie physique et d'optique, et Centre de recherche sur le cancer Université Laval Québec Québec Canada; ^3^ Service de physique médicale et de radioprotection, Centre intégré de cancérologie CHU de Québec‐Université Laval et Centre de recherche du CHU de Québec Québec Canada

**Keywords:** brachytherapy, electromagnetic tracking, HDR, plastic scintillation dosimeter

## Abstract

**Purpose:**

To evaluate the performance of an electromagnetic (EM)‐tracked scintillation dosimeter in detecting source positional errors of IVD in HDR brachytherapy treatment.

**Materials and Methods:**

Two different scintillator dosimeter prototypes were coupled to 5 degrees‐of‐freedom (DOF) EM sensors read by an Aurora V3 system. The scintillators used were a 0.3 × 0.4 × 0.4 mm^3^ ZnSe:O and a BCF‐60 plastic scintillator of 0.5 mm diameter and 2.0 mm in length (Saint‐Gobain Crystals). The sensors were placed at the dosimeter's tip at 20.0 mm from the scintillator. The EM sampling rate was 40/s while the scintillator signal was sampled at 100 000/s using two photomultiplier tubes from Hamamatsu (series H10722) connected to a data acquisition board. A high‐pass filter and a low‐pass filter were used to separate the light signal into two different channels. All measurements were performed with an afterloader unit (Flexitron‐Elekta AB, Sweden) in full‐scattered (TG43) conditions. EM tracking was further used to provide distance/angle‐dependent energy correction for the ZnSe:O inorganic scintillator. For the error detection part, lateral shifts of 0.5 to 3 mm were induced by moving the source away from its planned position. Indexer length (longitudinal) errors between 0.5 to 10 mm were also introduced. The measured dose rate difference was converted to a shift distance, with and without using the positional information from the EM sensor.

**Results:**

The inorganic scintillator had both a signal‐to‐noise‐ratio (SNR) and signal‐to‐background‐ratio (SBR) close to 70 times higher than those of the plastic scintillator. The mean absolute difference from the dose measurement to the dose calculated with TG‐43U1 was 1.5% ±0.7%. The mean absolute error for BCF‐60 detector was 1.7% ±1.2% when compared to TG‐43 calculations formalism. With the inorganic scintillator and EM tracking, a maximum area under the curve (AUC) gain of 24.0% was obtained for a 0.5‐mm lateral shift when using the EMT data with the ZnSe:O. Lower AUC gains were obtained for a 3‐mm lateral shifts with both scintillators. For the plastic scintillator, the highest gain from using EM tracking information occurred for a 0.5‐mm lateral shift at 20 mm from the source. The maximal gain (17.4%) for longitudinal errors was found at the smallest shifts (0.5 mm).

**Conclusions:**

This work demonstrates that integrating EM tracking to in vivo scintillation dosimeters enables the detection of smaller shifts, by decreasing the dosimeter positioning uncertainty. It also serves to perform position‐dependent energy correction for the inorganic scintillator,providing better SNR and SBR, allowing detection of errors at greater distances from the source.

## 1 INTRODUCTION

High‐dose‐rate (HDR) brachytherapy is a radiation therapy procedure in which a radioactive source is moved through multiple locations in a tumor site. There is a high dose gradient in the vicinity of an ^192^Ir brachytherapy source: at 10 mm from the source dose can differ by more than 20%/mm. Therefore a small shift of the source position can lead to large dosimetric discrepancies in the dosimetry. During HDR treatment, comparisons of plans' dwell times, dwell positions and measured doses to those of an expected plan can be used as error indicators. Error detection and treatment verification in brachytherapy can be performed by real‐time in vivo dosimetry (IVD).^1^ For this purpose, detector needs to be small and have a high dynamic range to be useful.^2^

Numerous works have investigated many different detectors for use in brachytherapy, as presented in a review of Tanderup et al.^2^ In particular, plastic scintillation dosimeters (PSDs) have been widely studied.^3^, ^4^, ^5^, ^6^ The absorption and scattering properties of PSDs match those of water, which facilitates the conversion from dose to scintillator to dose to tissue. Some studies have also shown the feasibility of using an inorganic crystal for this purpose. Some of the advantages of inorganic scintillation detectors (ISDs) over organic scintillators include higher scintillation light yield and lower stem contribution. A study by Kertzscher and Beddar showed a high scintillation light yield with an ISD 250 times that of a BCF‐12 plastic scintillator.^7^

A review of clinical brachytherapy uncertainties demonstrated that for many cancer sites, inter‐ and intra‐fraction changes were responsible for the highest percentage of dosimetric uncertainty.^8^ For IVD, one cause of dose uncertainties is the difficulty of knowing the exact position of the dosimeter during dose delivery. During this step, there is no visual available in real‐time to ensure the dosimeter location. The use of a tracking technology could solve this ambiguity.^1^

It was previously shown that inorganic scintillators have higher light yields than organic scintillators.^7^ Despite the advantage for higher signals far from the source, depth and angulation dependent energy correction factors are needed. Once again, a tracking technology could be used to relay angular and positional information of an inorganic scintillation dosimeter with accuracy, leading to real‐time application of the appropriate correction factors. Thus, an efficient tracking technology solves the two major shortcomings of scintillation dosimeters for IVD in HDR brachytherapy.^1^, ^7^, ^9^

In this study, we quantified the impact of adding real‐time tracking capability on error detection with in vivo dosimeters. For this purpose, an electromagnetic (EM) tracking system is used. To our knowledge, this addition to IVD was not quantified for error detection in previous work. The EM system exploits a field generator that produces an inhomogeneous EM field, allowing a passive EM sensor (induction coils) to be tracked with 5 or 6 degrees‐of‐freedom (DOF) when it is placed within the field generator's active volume. This system has been widely used clinically to track surgical tools.^10^, ^11^ It also has been proposed^12^, ^13^, ^14^ and investigated for real‐time guidance of catheters in HDR Ir‐192 brachytherapy.^15^, ^16^, ^17^

We compared two prototype detectors, a PSD and an ISD, coupled to an EM sensor (with intrinsic submillimeter positional accuracy) for real‐time dosimeter position tracking. For both detectors, different calibration methods were used.^7^, ^18^

We evaluated the agreement of the dose measurements with the expected dose calculated with TG‐43. Finally, we compared error detection performance with and without EM tracking to determine the gain from using positional tracking information. This paper aims to demonstrate that EMT can improve precision of scintillation dosimeters used for IVD in the presence of larger dose gradients, such as HDR brachytherapy.

## 2 METHODS

### 2.1 Detectors' construction

**FIGURE 1:**
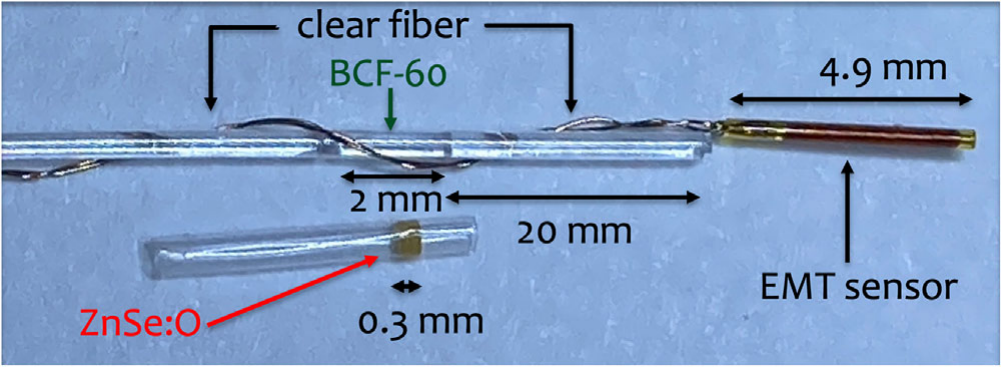
Construction of the dosimeter. The clear fiber between the BCF‐60 and the EMT sensor is not shown to scale in order to capture the entire dosimeter inside the microscope's field of view. EMT, electromagnetic tracking.

First, a PSD was constructed using BCF‐60 scintillators from Saint‐Gobain Crystals (Hiram, Ohio, USA) with a length of 2.0 mm and a diameter of 0.5 mm. A cubic ISD was constructed using an inorganic ZnSe:O scintillator of 0.3 × 0.4 × 0.4 mm^3^ (ISMA, Ukraine). Both detectors were coupled to an EM sensor with 5 DOF (Part Number 610157, NDI, Ontario, Canada) read by the Aurora V3 system (NDI, Ontario, Canada)^19^ (Figure 1). A space of 20.0 mm between the scintillator and the sensor was provided to ensure minimal influence of the EM sensor on the scintillation signal, as per Tho and Beaulieu.^19^ A collecting clear fiber of 0.5 mm diameter was used as a light guide. Multiple layers of heatshrink‐protecting tubes from Nordson Medical (Westlake, Ohio, USA;103‐0265, 103‐0007,103‐0447,103‐0159,103‐0081) were used to assemble the prototypes and ensure that they fit inside a 6F catheter used in HDR brachytherapy. The sensor was placed inside a polyetheretherketone plastic tube for additional protection. An external field generator producing an inhomogeneous EM field was used to generate induction signals in the sensor, providing the necessary spatial and angular tracking information within a specific volume.^11^ The sampling rate was 40 Hz. All measurements were done in a water tank of 40 × 40× 50 cm^3^. Solid water slabs were added on sides and bottom to ensure that TG‐43 full scatter conditions are met.

### 2.2 Light collection

The scintillation light was detected through a photomultiplier tubes (PMT) (Hamamatsu, Shizuoka, Japan; series H10722‐210) coupled to a dichroic mirror (series A10034) and filters (series A10033) to obtained two different spectral bands. The PSD signal was separated using a high‐pass filter of 496 nm and a low‐pass filter of 475 nm for the chromatic removal technique.^18^ A high‐pass filter of 519 nm and a low‐pass filter of 475 nm were used for the ISD. The data acquisition was performed using a NI USB‐6289 M Series Multifunction I/O device (National Instruments, Austin, Texas, USA) with a sampling rate of 100 000 Hz. The signal was averaged over 1000 samples, thereby producing 100 data points per second. All measurements were performed using a Flexitron afterloader unit (Elekta Brachy, Veenendaal, The Netherlands) in full TG‐43 scattered conditions.^20^

### 2.3 Calibration and energy dependence

The detectors were moved to different source‐dosimeter distances ranging from 8 to 70 mm by using a robotic arm (MECA500, Mecademic Robotic, Montreal, Canada). The robotic arm's positional uncertainty is 6 μm.^21^ To quantify the afterloader positional uncertainty, five measurements using a ruler after and before an experimental session were performed. EMT data were used to compute the standard deviation as well in a setup without the metallic ruler. The detector's position was continuously tracked with the Aurora system while the detector was moving. The calibration was performed with the method proposed by Guillot et al. for the BCF‐60 PSD prototype with a two‐channel signal.^18^ For the ZnSe:O ISD, a correction for the energy dependence with distance (in water) was needed. Only the signal that passed through the high‐pass filter was analyzed in this case, as the Cerenkov was negligible.^7^ The ratio of that signal to the dose calculated with TG‐43 formalism was normalized to the value at a distance of 20 mm from the source to be consistent with prior results using this scintillator. Interpolation in a grid of measurement was used for calibration as suggested by Jorgensen et al.^9^

Signal‐to‐noise ratio (SNR) and signal‐to‐background (SBR) ratio were also computed for both constructions for comparison. The SNR was computed with the mean of a dwell measurement on the standard deviation (SD) of that dwell measurement. The SBR was computed with the mean of a dwell measurement on the mean of the background measurement.

### 2.4 Error detection

**FIGURE 2:**
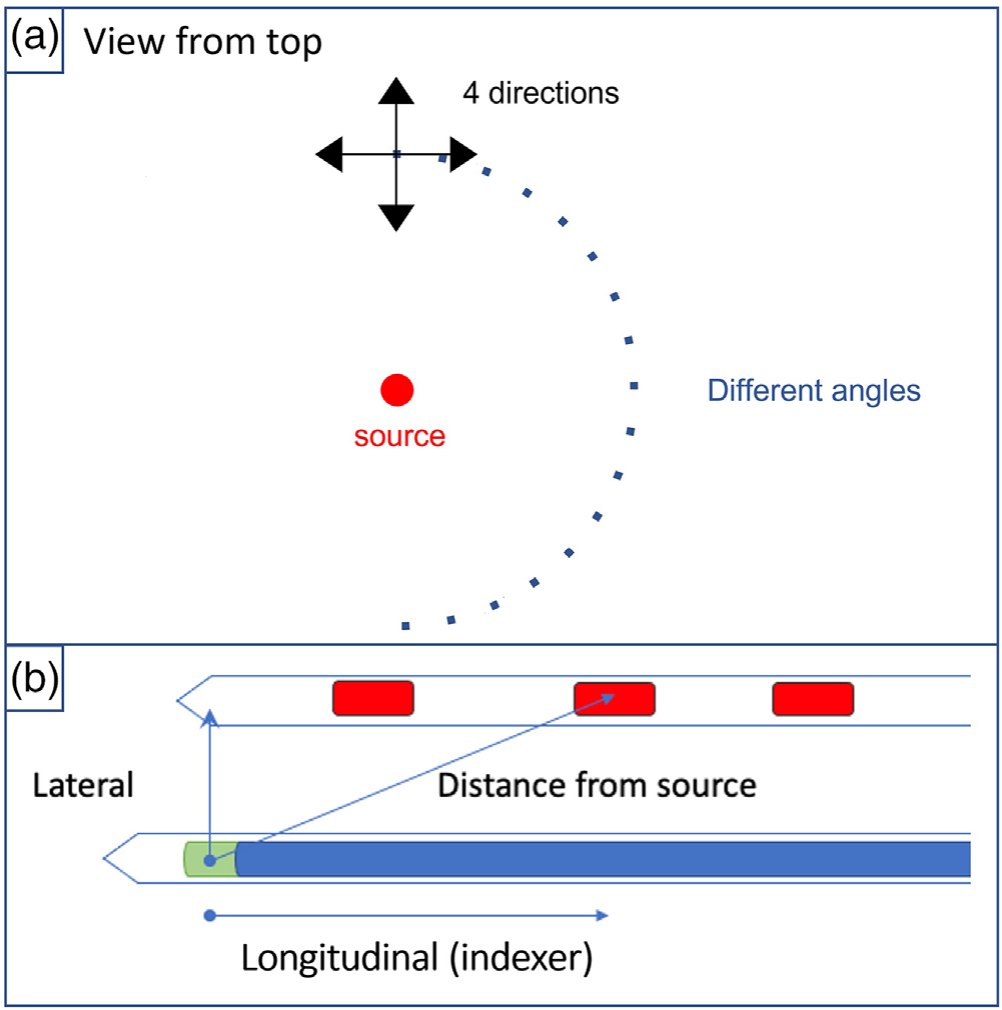
Representation of the water tank top view and the shift's geometry. (a) The 18 different angles and the four directions used for all shifts at every source‐dosimeter distances are shown. (b) Catheter with various active dwell positions and a second catheter containing a scintillation dosimeter.

Lateral and longitudinal shifts are defined in Figure 2b. Known lateral shifts of 0.5, 1, 2 and 3 mm were introduced at different distances from the source ranging from 20 to 70 mm in increments of 10 mm. Indexer's (longitudinal) shifts of 0.5, 1, 5, and 10 mm were placed at various distances, also ranging from 20 to 70 mm. These shifts were chosen from previous works based on their dosimetric impact during brachytherapy treatments.^1^, ^2^, ^22^ For error detection purposes, differences in dose rates were assumed to be caused by shifts in source‐dosimeter distance, as mentioned in Johansen et al.^23^ Receiver operating characteristic (ROC) curves were generated, and the areas under the curve (AUCs) were reported with and without use of the real‐time EM tracking of the dosimeter position. When tracking information was not used, the dosimeter's expected nominal (geometric) position was assumed. For each shift at a specific distance from the source, four measurements were acquired at a precise angle. 18 angles were chosen for these measurements (Figure 2a). Each ROC curve generated had at least 9 data points.

## 3 RESULTS

### 3.1 Calibration and absorbed dose measurements

**FIGURE 3:**
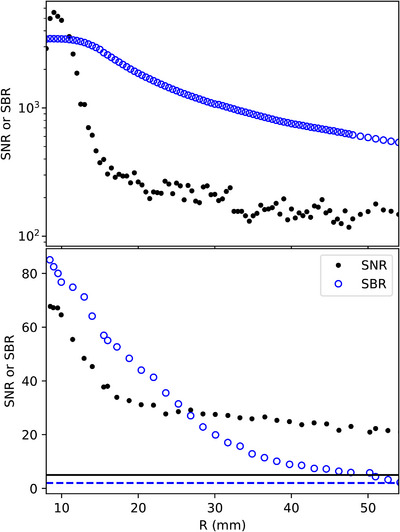
SBR and SNR for the inorganic ZnSe:O scintillator (top) and the plastic BCF‐60 scintillator (bottom). The dashed line corresponds to an SBR of 2 which is the detectability limit. The solid line corresponds to an SNR of 5 which is the Rose criteria. SBR, Signal‐to‐background ratio; SNR, signal‐to‐noise ratio.

The afterloader used for the experiment showed a maximum afterloader position variation of 1.0 mm. The standard deviation of a fixed position computed with the EMT data was 0.6 mm. Figure 3 shows the SNR and the SBR for both scintillators. The ISD had ratios about 70 times higher than those of the BCF‐60 PSD. The SBR of the PSD dropped below the detectability limit of 2 when the source‐detector distance reached 60 mm, which was not the case for the ZnSe:O ISD.

**FIGURE 4:**
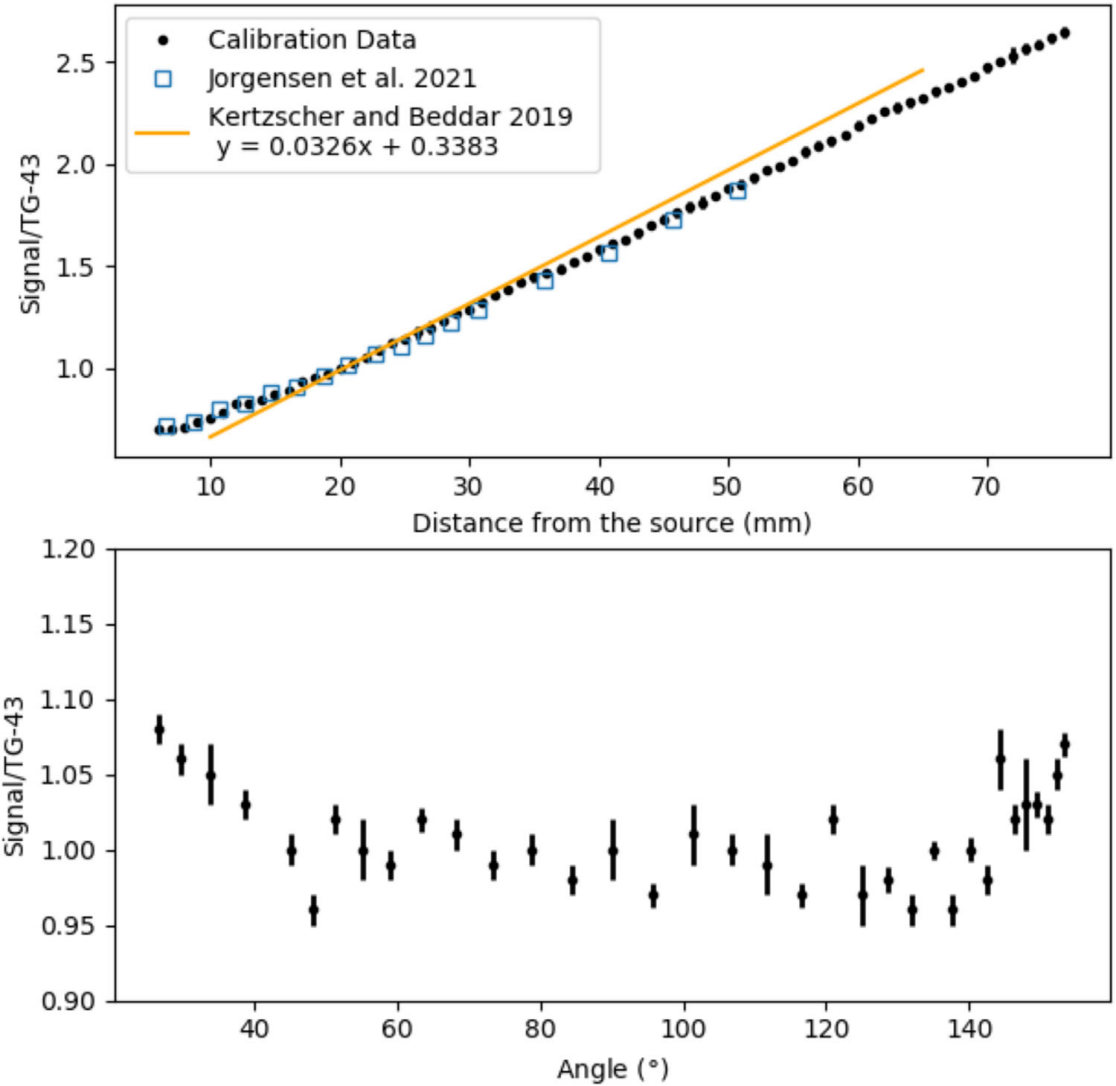
Distance (top) and angular (bottom) dependency of the ZnSe:O ISD. The ratios were normalized to the value at a distance of 20 mm from the source. The yellow line correspond to the calibration linear fit (range 8–67 mm) from a paper of Kertzscher and Beddar.^7^ The blue squares represent the values from Jorgensen et al.^9^ ISD, inorganic scintillation detector.

Figure 4 presents the correction needed for the ZnSe:O ISD according to the distance and angle from the source. There was a slight angular dependency when the detector was further from 90°.

**FIGURE 5:**
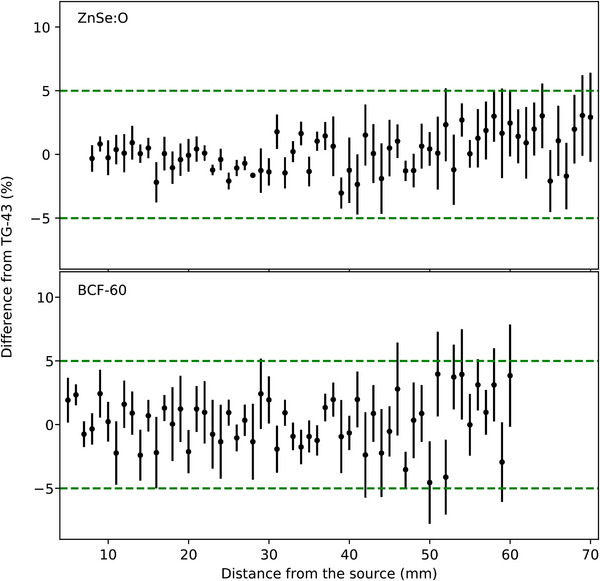
Differences with TG‐43 for the ZnSe:O ISD (top) and the BCF‐60 PSD (bottom). The dashed green lines correspond to the ±5% difference from TG‐43 calculations.

With the calibration completed for both detectors, their ability to measure known doses in TG43 conditions was tested. Figure 5 shows the difference between dose calculated with TG‐43 formalism and the measurement for each dosimeter at multiple distances from the source. Over the 8‐ to 60‐mm source‐detector distance range, the mean absolute error for the ZnSe:O ISD was 1.5% ± 0.7%. The mean absolute error for BCF‐60 PSD was 1.7% ± 1.2% over the same distance range. It is worth noting that the larger standard deviation (variability) for the PSD can be attributed to the lower SNR and SBR with distance from the source.

### 3.2 Error detection

**FIGURE 6:**
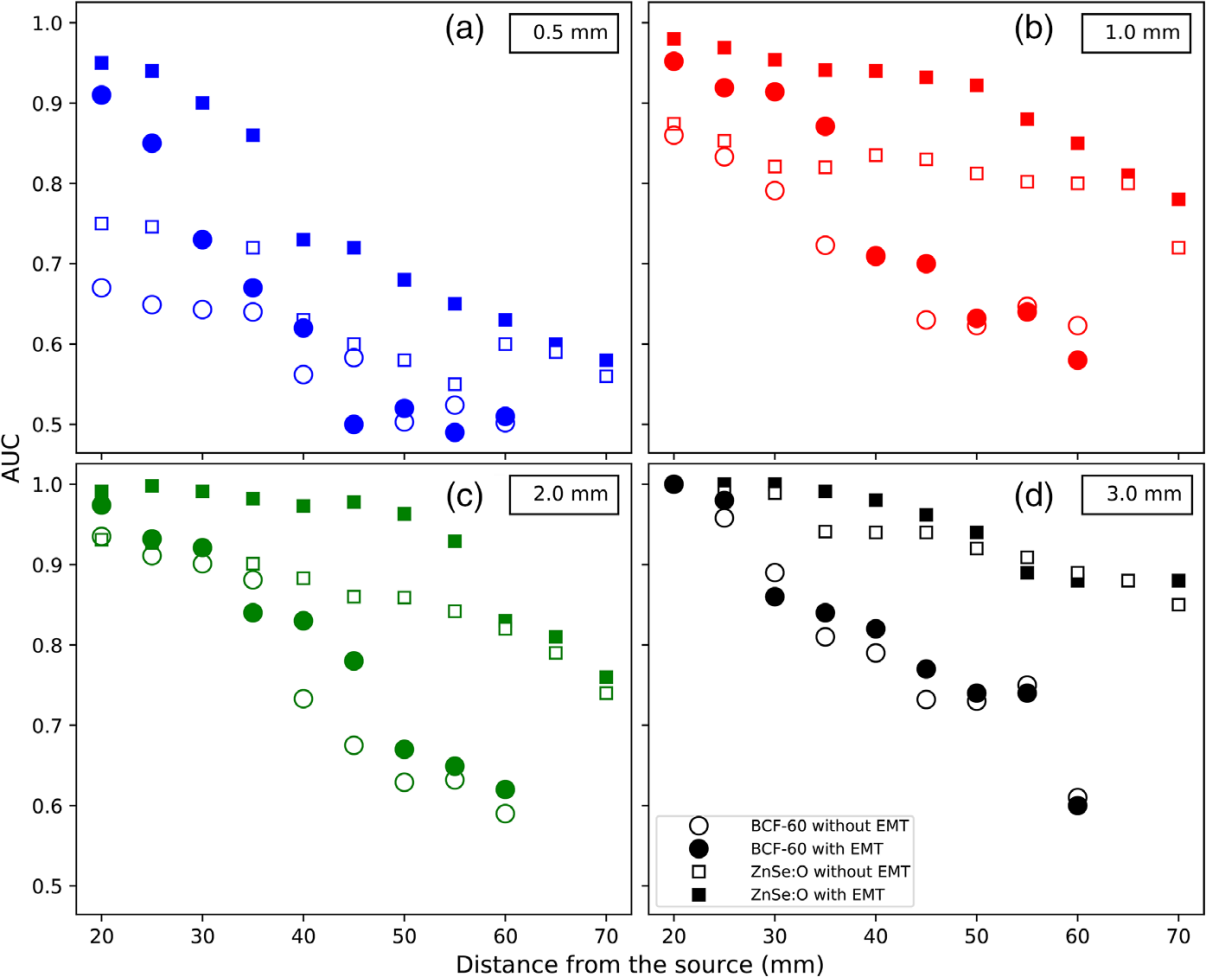
AUC as a function of lateral shifts of 0.5 (a), 1.0 (b), 2.0 (c) and 3.0 mm (d). Circles (filled and open) indicate the BCF‐60 PSD, and squares (filled and open) indicate the ZnSe:O ISD. Filled symbols correspond to the AUCs for the detectors located by EMT, while open symbols correspond to the baseline AUCs without EMT. AUC, Area under the curve; EMT, EM tracking; ISD, inorganic scintillation detector; PSD, plastic scintillation dosimeters.

Figure 6 shows the AUCs for lateral shifts of 0.5 , 1.0 , 2.0 , and 3.0 mm. The mean gain offered by tracking the dosimeters for all shifts was 5.6%. However for a 0.5‐mm shift, the gain from dosimeter tracking was as high as 24.0%. The PSD had its highest gain using EM at 20 mm from the source for a 0.5‐mm lateral shift. With a 1‐mm shift, the maximum gain decreased to 14.8% and decreased even more with a 2‐mm shift to 11.8%. For a 3‐mm shift, the gain was higher for the ISD (5.0%) than for the PSD (3.8%). For larger shifts, the gain provided by the EMT system decreased, becoming negligible beyond 55 mm, even for the smaller 0.5 and 1.0‐mm shifts.

**FIGURE 7:**
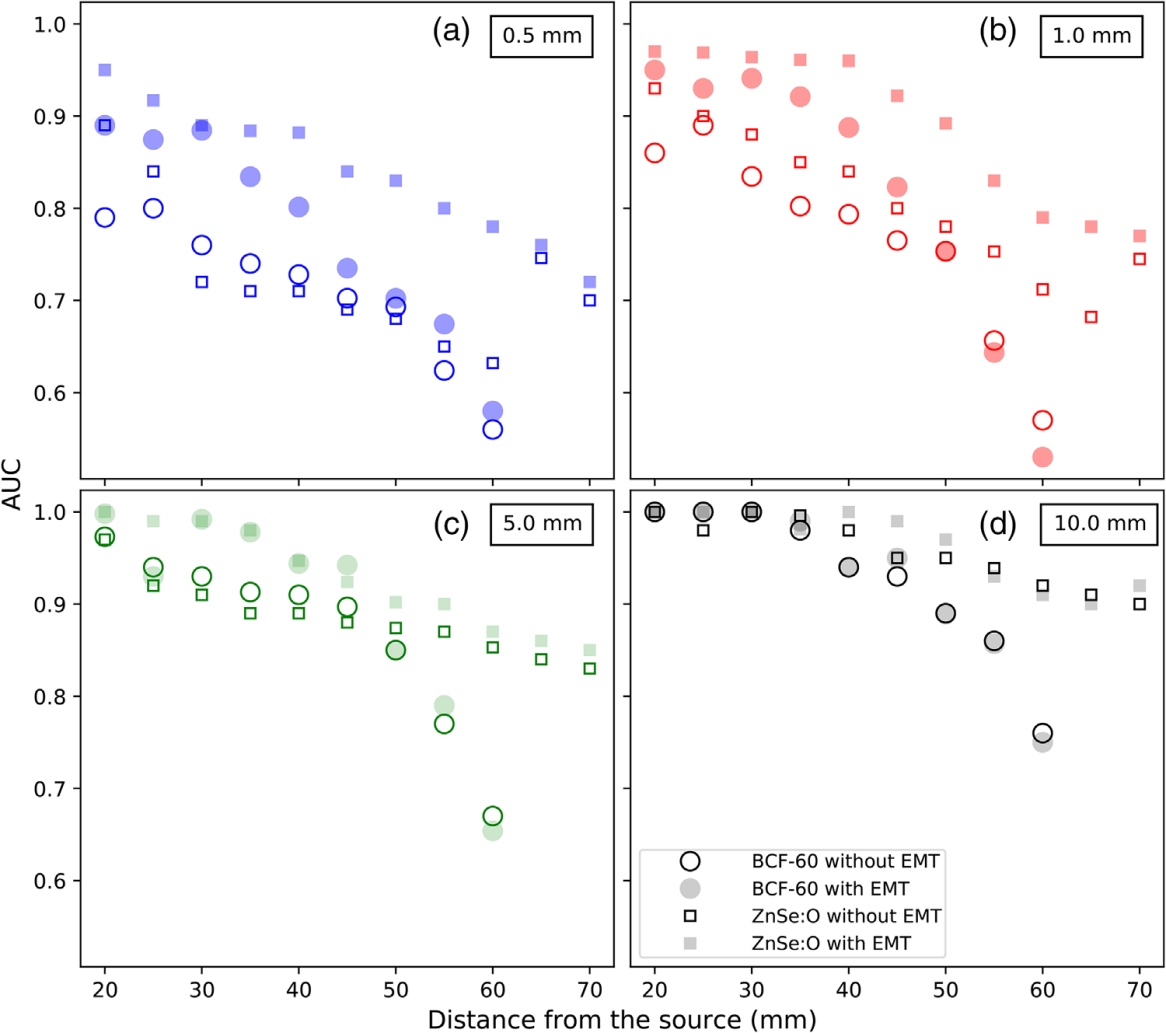
AUC as a function of longitudinal shifts of 0.5 (a), 1.0 (b), 5.0 (c), and 10.0 mm (d). Circles (filled and open) indicate the BCF‐60 PSD, and squares (filled and open) indicate the ZnSe:O ISD. Filled symbols correspond to the AUCs for the detectors located by EMT, while open symbols correspond to the baseline AUCs without EMT. AUC, Area under the curve; EMT, EM tracking; ISD, inorganic scintillation detector; PSD, plastic scintillation dosimeters.

Next, we calculated the AUCs for the indexer length errors (Figure 7). For longitudinal shifts, the maximum gain with EMT, 17.4%, occurred at 35 mm from the source for a 0.5‐mm shift. The mean gain for this shift was 9.3% over all distances and 10.9% for distances under 40 mm. Similar to the lateral shifts, the lowest gain with EM tracking was observed for the largest (5‐ and 10‐mm) indexer errors. At 10 mm, there was no gain, as large shifts occurring within a 30‐mm source‐dosimeter distances can be detected to almost 100% accuracy by conventional, untracked dosimeters. Note that for the ZnSe:O ISD, the use of the tracking information always gave a higher AUCs over the complete range of distances for shifts of 0.5 and 1 mm, with the maximum gain at 17.4%.

## 4 DISCUSSION

The benefits of using EM tracking data were larger when the detector was closer to the source and for small displacements. Accurate determination of the dosimeter location is critical for small dosimeter‐source distances because of the strong dose gradient at those distances; small shifts lead to large dose discrepancies. For 0.5‐mm lateral shifts, the use of EM tracking with the ISD always gave a higher detection rate for distances under 40 mm from the source. Here, we demonstrated that the use of an EM tracking system can detect small shifts in dosimeter positioning and decrease positional uncertainty close to an HDR brachytherapy source.

Inorganic scintillators require energy correction, which leads to higher uncertainties when the source‐detector distance increases. Our calibration data for the ISD were similar to those presented by Jorgensen et al.^9^ However, a previous characterization of the same scintillator by Kertzscher and Beddar^7^ found a slightly different behavior. The difference could be explained by the different amount of data used for the calibration in the study. The EM tracking information was able to provide the necessary information to correct the ISD energy and angular dependencies, as demonstrated in Figure 4. The ISD had a higher SNR and SBR ratios than did the PSD. Thus, the ISD's sensitivity was adequate to meet the detectability limit (SBR > 2) at source‐detector distances over 60 mm. The SNR signal was sufficient (SNR > 5) for both detectors at a distance over 60 mm to meet the Rose criteria for detection.^24^ However, for both detectors, the differences from TG‐43 measurements became greater when they were positioned further from the source, with more variability for the PSD owing to its lower signal generation.

This study also demonstrated that at larger source‐detector distances, the usefulness of dosimeter tracking is not always obvious, and tracking may often not be needed. In particular, for lateral shifts, the use of EM tracking with ISD appears more useful over a larger range of shifts and source‐dosimeter distances compared to PSD. This can be explained by the larger signal (SNR and SBR) of the ISD and also by the use of the EM tracking positioning information to correct the energy dependence of the ISD. For large lateral shifts, that is over 3 mm, dosimeter tracking information provided essentially no advantages.

One limitation of this study is that we did not account for the temperature dependence of the system if used inside a patient. Many studies have shown that these scintillators have a non‐negligible temperature dependence.^9^, ^25^ In this study, the calibration and measurements were performed in a water tank with well‐controlled conditions and therefore, temperature change was not an issue. Another limitation was the minimum source‐detector distance of 20 mm used. Due to the high dose gradients involved in HDR brachytherapy, adding positional information for detecting shifts is even more important closer to the source. When making the required displacement, the robotic arm Meca500 does not always take a linear path (due to its rotational joints); for this reason a minimal approach distance was needed to ensure that there would not be a collision between the robot and the source holder or the transfer tube. For the extended reach needed, using a simple joint of the robot was not possible.

While this work demonstrates the usefulness of EM tracking in IVD to provide knowledge of the dosimeter location and to allow for distance and angulation correction factors of energy dependent dosimeters, more work is needed to understand the action threshold for clinical intervention based on the measurements provided by such a system. Furthermore, a robust software integration that introduces all this new information with the standard treatment parameters needs to be available for efficient clinical use.

## 5 CONCLUSIONS

We explored the need for real‐time tracking of dosimeter position during IVD for two scintillation dosimeters coupled with EM sensors. The inorganic scintillator had higher sensitivity, which made it possible to reliably measure dose at a greater distance from the source by using EM tracking information to provide energy and angular dependence corrections. Adding EM tracking was shown to greatly improve the detection of errors caused by small shifts close to the source (⩽ 30 mm), where the dose gradient is large. The use of a tracking technology thus efficiently solves the ambiguity of the dosimeter location in IVD for brachytherapy. Overall, the use of EMT reduces the false‐positive rate, resulting in higher AUCs by providing the dosimeter location in real time.

## CONFLICT OF INTEREST STATEMENT

The authors declare no conflicts of interest.
